# Prognostic significance of microRNA-135 in patients with digestive system cancers: a systematic review and meta-analysis

**DOI:** 10.1042/BSR20190845

**Published:** 2019-12-16

**Authors:** Ce Chao, Chen Sang, Min Wang, Zijin Wang, Yanfei Li, Guanghua Luo, Xiaoying Zhang

**Affiliations:** 1 Department of Cardiothoracic Surgery, The Third Affiliated Hospital of Soochow University, Changzhou 213003, China; 2 Comprehensive Laboratory, The Third Affiliated Hospital of Soochow University, Changzhou 213003, China

**Keywords:** digestive system cancer, meta analysis, microRNA-135, prognosis

## Abstract

**Background:** MicroRNA-135 (miR-135) is a well-known non-coding RNA that has been demonstrated to participate in tumorigenesis and cancer development; however, the clinical prognostic value of miR-135 in digestive system cancers remains controversial. This meta-analysis aims to explore the potential value of miR-135 as a prognostic marker for digestive system cancers.

**Methods:** The PubMed, Embase, Cochrane Library, and Web of Science databases were searched for eligible articles published before 31 August 2019. Stata 12.0 software was used to analyze the overall survival (OS), disease-free survival (DFS), and recurrence-free survival (RFS) rates to access the prognostic value of miR-135 in digestive system cancers. We then used The Cancer Genome Atlas (TCGA) datasets to validate the meta-analysis results.

**Results** A total of 1470 patients from 17 studies were included in this meta-analysis. The pooled results showed that enhanced miR-135 expression was significantly associated with poor OR (hazard ratio (HR): 1.790; 95% confidence interval (95% CI): 1.577–2.031; *P*=0.000), DFS (HR: 1.482; 95% CI: 0.914–2.403; *P*=0.110), and RFS (HR: 3.994; 95% CI: 1.363–11.697; *P*=0.012) in digestive system cancers. A sensitivity analysis confirmed the reliability of our findings, and no significant publication bias was observed.

**Conclusion:** MiR-135 can be used as a novel biomarker for patients with digestive system cancers. We look forward to future large-scale clinical studies that will investigate the prognostic value of miR-135.

## Introduction

Digestive system cancers are one of the most common malignancies throughout the world. Such digestive system cancers include esophageal, gastric, colorectal, liver, gallbladder, and pancreatic cancers (PCs). According to the latest reports, there are approximately 4.9 million new diagnosed cases and 3.5 million deaths for digestive system cancers that occur worldwide each year [1]. Although surgical techniques and immunotherapy have been developed, the prognosis of patients with digestive system cancers remains poor. With increased in-depth research into long non-coding RNA, miRNA, and epigenetics, we have gained a better understanding of cancer at the genetic level [2–4]. Therefore, we expect to identify reliable biomarkers that can be used as prognostic factors from these in digestive system cancers. Recently, miRNAs have also attracted increased attention and are considered to be potential biomarkers for cancer prognosis [5,6].

The iRNAs are short non-coding RNAs (ncRNAs) comprising approximately 18−23 nucleotides that mediate gene silencing by guiding Argonaute (AGO) proteins to target sites in the 3′ untranslated region (UTR) of mRNAs [7]. Since their serendipitous discovery in nematodes, miRNAs have emerged as key regulators of various biological processes in animals [8]. To date, over 30000 miRNAs have been identified. Moreover, miRNAs have been applied to both basal experimental studies, as well as Phase I clinical trials for treating cancer [9]. MicroRNA-135 (MiR-135) is a member of the miRNA family that plays an important role in a variety of cancers, and has two isoforms, miR-135a and miR-135b. Although these isoforms are located on different chromosomes, mature miR-135a and miR-135b only differ in one nucleotide, which is not within the miRNA binding region. Thus, it has been reported that both miRNAs target the same genes [10,11].

Studies have reported that miR-135 is overexpressed in several human cancers and cancer cell lines, including PC [12], breast cancer [13], colorectal cancer (CRC) [14], gastric cancer [15], among others. However, the relationship between miR-135 expression and digestive system cancers remains controversial. Despite this discrepancy, an increasing number of recent studies have emphasized the role of miR-135 in predicting poor tumor prognosis. In addition, based on a bioinformatics analysis, miR-135 was considered worthy of further investigation as a potential novel biomarker for the diagnosis and prognosis of esophageal squamous cell carcinoma (ESCC) and PC [16,17]. In contrast, the prognostic value of miR-135 in gastric cancer was associated with the opposite result [18]. Therefore, the aim of the present study was to evaluate the association of miR-135 expression with the overall survival (OS), disease-free survival (DFS), and recurrence-free survival (RFS) of patients with digestive system cancers.

## Methods

### Search strategy

In this meta-analysis, the PubMed, Embase, Web of Science, and Cochrane Library databases were systematically searched using: ‘microRNA 135 OR microRNA 135a OR microRNA 135b OR miRNA 135 OR miRNA 135a OR miRNA 135b OR miR-135 OR miR-135a OR miR-135b OR miR-135a-3p OR miR-135a-5p OR miR-135b-3p OR miR-135b-5p’ and ‘tumor or cancer or carcinoma or neoplasm’ for studies published up to 31 August 2019. For the Cochrane Library, we searched the keywords ‘microRNA or miRNA’ and ‘tumor or cancer or carcinoma or neoplasm’ to avoid missing suitable studies. Moreover, the reference of eligible studies and review articles was also manually reviewed for suitable studies. While searching and screening the eligible studies, the Preferred Reporting Items for Systematic Review and Meta-Analysis (PRISMA) [19] and Assessing the Methodological Quality of Systematic Reviews (AMSTAR) Guidelines were followed.

### Inclusion and exclusion criteria

The inclusion criteria were as follows: (1) the studies researched digestive system cancers; (2) the studies investigated the association between miR-135 and clinical prognosis; (3) the study provided available and usable data for estimating the hazard ratios (HRs) and 95% confidence intervals (95% CIs) for survival. Furthermore, studies were excluded if they conformed to one of the following criteria: (1) insufficient data to estimate the prognostic correlation; (2) basic medical experiment, animal studies, case reports, and review articles; and (3) duplicated data.

### Quality assessment

Two investigators (Ce Chao andChen Sang) independently scored the included studies according to the Newcastle–Ottawa Quality Assessment Scale [20]. The final scores were presented from 0 to 10, with higher scores indicating better methodological quality.

### Data extraction

Two investigators (Min Wang and Yanfei Li) independently extracted all the data from eligible studies. The following data were extracted: the first author, year of publication, type of cancer, size of sample, country of origin, follow-up time, detection methods, treatment measure, patient survival, and statistical methods. If the survival data were not reported, it was extracted from Kaplan–Meier curves for further calculation. Finally, the third investigator was consulted to review all the data .

### Extraction and analysis of The Cancer Genome Atlas datasets

The level of miR-135 expression and clinical data for The Cancer Genome Atlas (TCGA) digestive system cancer (including cholangiocarcinoma, colon adenocarcinoma, esophageal carcinoma, liver hepatocellular carcinoma (HCC), pancreatic adenocarcinoma, rectum adenocarcinoma, and stomach adenocarcinoma) were extracted from the UCSC Xena website (https://xenabrowser.net/hub/) [21]. The level of miRNA expression was measured using the IlluminaHiseq platform. There were 1488 patients with digestive system cancers who had both survival and miR-203 expression data in the TCGA Pan-cancer datasets for this subject [22]. The level of miR-135 expression was distinguished based on receiver operating characteristic (ROC) curves with a Youden’s index correction [23]. The survival curves were plotted and tested using the Kaplan–Meier method and a log-rank test using Stata software version 12.0 (Stata Corporation, College Station, TX).

### Statistical analysis

In this meta-analysis, all pooled results were analyzed with Stata software version 12.0 (Stata Corporation, College Station, TX). Pooled HRs with the corresponding 95% CIs were used to estimate the strength of the association between miR-135 and the clinical prognosis of cancer patients. The optimal method was to obtain survival data from the articles or calculate the HRs from O-E statistics and variance. Otherwise, Kaplan–Meier curves were analyzed using the Engauge Digitizer version 4.1 according to the method introduced by Parmar et al. [24]. The level of heterogeneity was assessed by the inconsistency index *I^2^* and χ^2^-based Q test. *I^2^* statistics with values >50% and a χ^2^ test with *P*<0.05 indicated a strong heterogeneity across the studies. If the heterogeneity was not significant, the fixed-effect model was appropriate; otherwise, the random-effect model was adopted. Subgroup analysis was conducted for tumor type, digestive function, biomarker subtype, publication year, sample size, the method of HRs extraction, and quality score. The meta-analysis results were displayed as forest plots. Sensitivity analysis was performed to test the impact of an individual study on the pooled data by omitting each single study. Begg’s funnel plots and an Egger’s linear regression test were performed to estimate any potential publication bias [25]. False-positive report probability (FPRP) analysis and trial sequential analysis (TSA) were performed to assess the significant associations [26,27].

## Results

### Study characteristics

**Figure 1:**
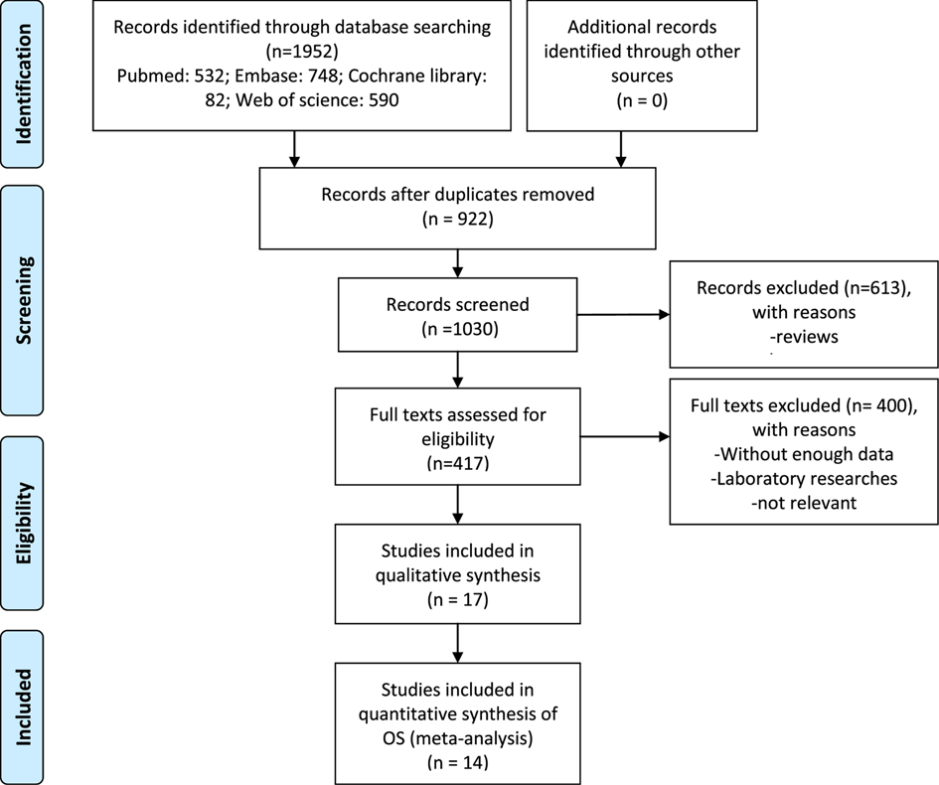
Flow chart for selection of studies for inclusion in this meta-analysis

A total of 1952 potentially relevant studies were retrieved from the electronic databases using the search strategy. After removing any duplications and carefully screening the abstracts and full-text of these articles, a total of 17 articles [14,15,18,28–41] were finally included in the current quantitative analysis of the prognostic value of miR-135 for digestive system cancers. The selection process of the literature search is presented in Figure 1. The general characteristics of these studies are summarized in Table 1. The 17 included studies were reports from around the world, including 14 from China [14,15,18,28,30,31,33,34,36–41], 1 from the U.K. [29], and 2 from Germany [32], all of which were published between 2012 and 2019. Among the included studies, five focused on CRC [14,29,30,35,37], four were on HCC [28,32,39,41], three were about gastric cancer [15,18,31], one was on ESCC [34], three were regarding PC [33,38,40], and one investigated gastric cardia adenocarcinoma (GCAC) [36]. There were 14 studies [14,15,18,28–31,33,34,36–38,40,41] involving 1225 patients that reported the OS, 7 [14,15,28,33,35,39,40] that reported DFS, and 2 [29,32] reported the RFS.

**Table 1:** Characteristics of the studies included in the meta-analysis

Author	Time	Country	Sample size	Cancer type	Quality	Follow-up	Methods	Treatment	Survival data	Subtype	Cut-off	Source of HR
Gaedcke	2012	Germany	116	RC	7	120	qPCR	Multiple	DFS, CSS	b	Median	Reported
Liu	2012	China	50	HCC	7	25	qPCR	Surgery	DFS, OS	a	Median	Reported
Valeri	2014	U.K.	62	CRC	5	NR	qPCR	Surgery	RFS, OS	b	Median	Curve
Gao	2016	China	58	GCAC	5	NR	qPCR	Surgery	OS	b	Median	Curve
Kan	2016	China	105	CRC	8	60	qPCR	Surgery	OS	b	Median	Reported
Li	2016	China	47	CRC	8	NR	qPCR	Multiple	OS	a	Mean	Curve
Yan	2016	China	280	GC	8	60	qPCR	Surgery	DFS, OS	a	Median	Reported
Cheng	2017	China	176	GC	6	60	qPCR	Surgery	OS	a	Mean	Curve
Han	2017	China	36	PC	8	NR	qPCR	Surgery	OS	b	Median	Reported
Huang	2017	China	103	HCC	8	NR	qPCR	Surgery	DFS	a	Normal	Curve
Felden	2017	Germany	26	HCC	8	NR	qPCR	Multiple	RFS	a	Median	Reported
Zhang	2017	China	85	PC	8	48	qPCR	Surgery	DFS, OS	b	Median	Curve
Jiang	2018	China	37	PC	6	NR	qPCR	Chemotherapy	OS, DFS	b	Mean	Curve
Qiao	2018	China	94	CRC	8	80	qPCR	Surgery	DFS, OS	a	Median	Curve
Zhang	2018	China	105	ESCC	6	NR	qPCR	Surgery	OS	-	Mean	Reported
Xie	2019	China	40	GC	5	102	qPCR	NR	OS	a	Median	Curve
Yang	2019	China	50	HCC	6	NR	qPCR	Surgery	OS	b	Normal	Curve

Abbreviations: CCS, cancer-specific survival; GC, gastric cancer; NR, not recorded; qPCR, real-time quantitative PCR detection system.

### Prognostic value of miR-135 for the OS of digestive system cancers

**Figure 2:**
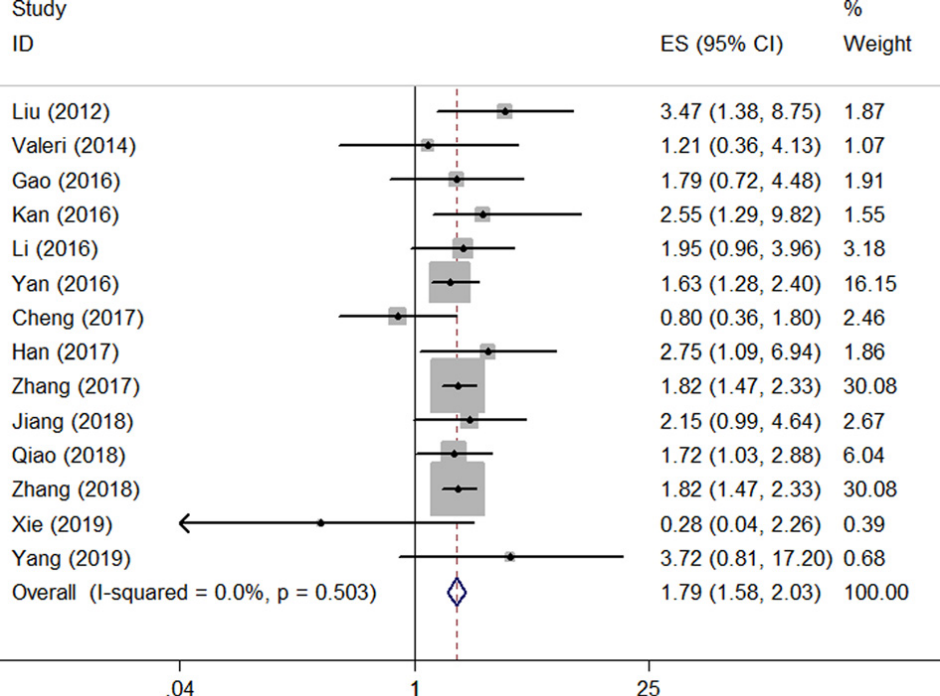
Meta-analysis of the pooled HRs of OS of patients with miR-135 overexpression

In this meta-analysis, a total of 14 studies, including a total of 1225 patients, reported HRs and associated 95% CIs for OS. As shown in Figure 2, the pooled results indicate that elevated miR-135 expression was associated with a poor OS in patients with digestive system cancer (HR: 1.790; 95% CI: 1.577–2.031; *P*=0.000). Moreover, a heterogeneity analysis revealed that there was no between-study heterogeneity in the eight studies (χ^2^ = 12.31; d.f. = 13; *P*=0.503; *I^2^* = 0.0%).

Although no inter-study heterogeneity was observed in this meta-analysis, a subsequent analysis was performed based on the cancer type, digestive function, biomarker subtype, publication year, sample size, the method of HR extraction, and quality score. As shown in Table 2, a significant correlation between miR-135 overexpression and a poor OS was shown in patients with HCC (HR: 3.536; 95% CI: 1.604−7.796; *P*=0.002), CRC (HR: 1.814; 95% CI: 1.257−2.619; *P*=0.001), PC (HR: 1.885; 95% CI: 1.521−2.336), and ESCC (HR: 1.820; 95% CI: 1.446–2.291). There was a significant association between miR-135 expression and the patient OS in the digestive gland (HR: 1.931; 95% CI: 1.591−2.341; *P*=0.000) and digestive tract (HR: 1.689; 95% CI: 1.428−1.997; *P*=0.000). Both miR-135a (HR: 1.624; 95% CI: 1.290−2.044; *P*=0.000) and miR-135b (HR: 1.902; 95% CI: 1.557−2.323; *P*=0.000) were significantly associated with poor OS in digestive system cancer patients. In addition, miR-135 was found to be significantly associated with the OS of patients reported in studies published from 2012 to 2016 (HR: 1.800; 95% CI: 1.403−2.309; *P*=0.000) and 2017 to 2018 (HR: 1.786; 95% CI: 1.543−2.068; *P*=0.000). The association between miR-135 and the OS of patients was significant in studies with a sample size less than 100 (HR: 1.879; 95% CI: 1.571−2.247; *P*=0.000) and more than 100 (HR: 1.706; 95% CI: 1.427−2.038; *P*=0.000). We also detected a significant relationship between the level of miR-135 expression and the OS of patients in both studies with a quality score less than 6 (HR: 1.717; 95% CI: 1.403−2.100; *P*=0.000) and those with a quality score greater than 6 (HR: 1.839; 95% CI: 1.563−2.162; *P*=0.000). Finally, a significant relationship was found between miR-135 and the OS of patients in both studies with HRs reported in the text (HR: 1.846; 95% CI: 1.548−2.202; *P*=0.000) and studies with HRs extracted from survival curves (HR: 1.732; 95% CI: 1.444–2.076; *P*=0.000).

**Table 2:** Results of subgroup analysis of pooled HRs of OS of patients with increased miR-135 expression

Stratified analysis	Number of studies	Number of patients	Heterogeneity		Pooled HR (95% CI)	Meta-regression *P*-value	Statistical power	FPRP *P*-value	FPRP
			*I^2^* (%)	*P*-value					
All	14	1225	0	0.503	1.790 (1.577–2.031)		-	0	-
Tumor type						-			
HCC	2	100	0	0.939	3.536 (1.604–7.796)		0.017	0.002	0.911
CRC	4	308	0	0.817	1.814 (1.257–2.619)		0.155	0.001	0.486
GC	3	496	61.5	0.075	1.031 (0.477–1.917)		0.882	0.923	0.990
PC	3	158	0	0.657	1.885 (1.521–2.336)		0.018	0.000	0.000
ESCC	1	105	-	-	1.820 (1.446–2.291)		0.050	0.000	0.001
GCAC	1	58	-	-	1.790 (0.718–4.465)		0.352	0.212	0.983
Digestive function						0.257			
Digestive grand	5	258	0	0.539	1.968 (1.600–2.421)		0.005	0.000	0.000
Digestive tract	9	967	0	0.442	1.692 (1.443–1.984)		0.069	0.000	0.000
Biomarker subtype						0.588			
miR-135a	6	687	43.1	0.118	1.624 (1.290–2.044)		0.249	0.000	0.014
miR-135b	7	433	0	0.874	1.902 (1.557–2.323)		0.010	0.000	0.000
Publication year						0.957			
2012–2016	6	602	0	0.666	1.800 (1.403–2.309)		0.076	0.000	0.005
2017–2019	8	623	22.9	0.247	1.786 (1.543–2.068)		0.010	0.000	0.000
Sample size						0.453			
<100	10	559	0	0.600	1.879 (1.571–2.247)		0.007	0.000	0.000
≥100	4	666	31.6	0.223	1.706 (1.427–2.038)		0.180	0.000	0.074
Quality score						0.603			
≤6	7	343	28.9	0.207	1.717 (1.403–2.100)		0.094	0.000	0.000
>6	7	556	0	0.732	1.839 (1.563–2.162)		0.007	0.000	0.000
HRs extraction						0.619			
Reported	5	675	0	0.478	1.846 (1.548–2.202)		0.011	0.000	0.000
Curve	9	649	6.5	0.381	1.732 (1.444–2.076)		0.060	0.000	0.000
DFS	7	765	86.9	0.000	1.482 (0.914–2.403)		0.520	0.111	0.995
RFS	2	88	0	0.879	3.994 (1.363–11.697)		0.037	0.012	0.969

Abbreviation: GC, gastric cancer.

**Figure 3:**
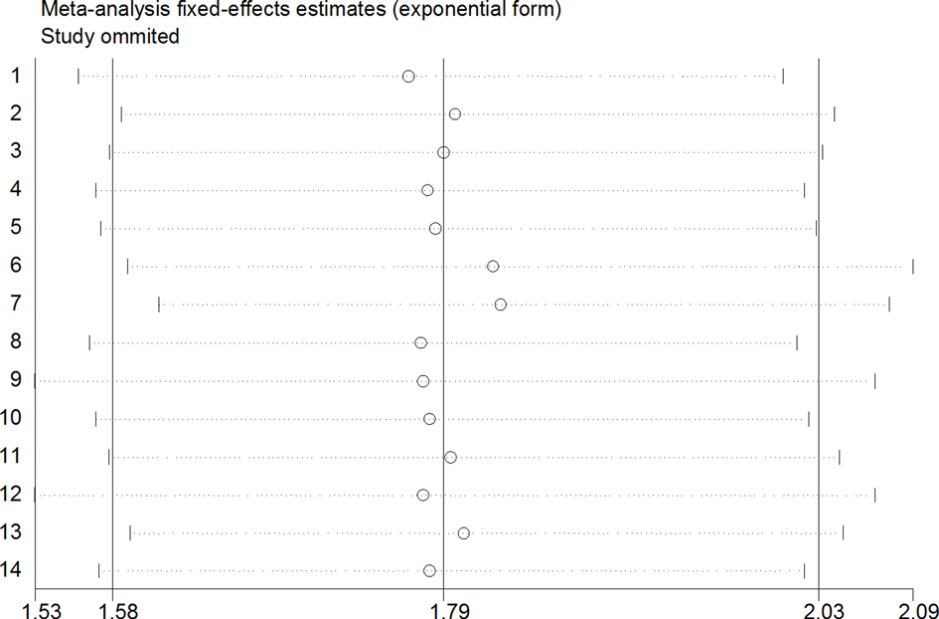
Sensitivity analysis of the effect of the individual study on the pooled HRs for the correlation between miR-135 expression and OS in patients with digestive system cancers

**Figure 4:**
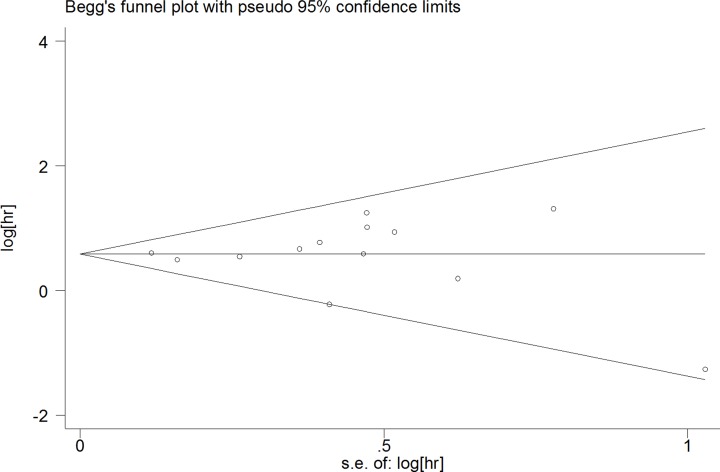
Begg’s funnel plot analysis for publication bias

A sensitivity analysis was performed to determine the reliability of the pooled results. The results proved to be solid by sequentially omitting each study (Figure 3). A Begg’s funnel plot and Egger’s test were conducted to assess the publication bias for the pooled OS. No publication bias was observed for the OS (Begg’s test: *P*=0.622; Egger’s test: *P*=0.932) (Figure 4).

### Prognostic value of miR-135 for the DFS and RFS of digestive system cancers

**Figure 5:**
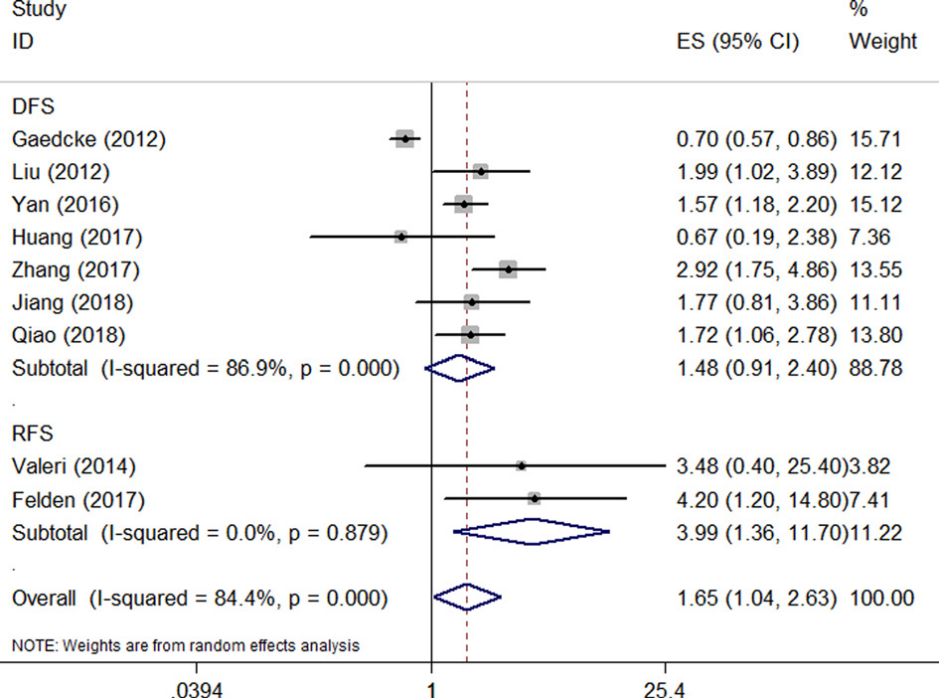
Meta-analysis of the pooled HRs of DFS and RFS of patients with miR-135 overexpression

Seven studies that included a total of 765 patients reported the level of miR-135 expression and DFS of digestive system cancers. Two studies consisting of 88 patients reported HRs with the 95% CI for RFS. As shown in Figure 5, miR-135 overexpression was associated with a poor RFS (HR: 3.994; 95% CI: 1.363–11.697; *P*=0.012), but DFS (HR: 1.482; 95% CI: 0.914–2.403; *P*=0.110) in patients with digestive system cancers.

### FPRP analysis

We performed an FPRP assay for OS, DFS, and RFS to uncover potential false-positive results among significant associations, with a prior probability of 0.01 and an FPRP cut-off value of 0.2. Because the *P*-value of FPRP for OS was equal to 0, the corresponding FPRP value was infinitely close to 0 [26]. Thus, there was a significant association between OS and digestive system cancer. Moreover, there was a significant association between OS and digestive system cancer observed in the subgroups of cancer type (PC and ESCC), cancer classification (digestive gland and tract), source of control (curve and reported), sample size (large and small), biomarker subtype (miR-135a and miR-135b), publication year (2012−2016 and 2017−2019), and quality score (more than 6 and less than 6). The FPRP values for DFS and RFS were all >0.2, revealing that these associations were not truly significant (Table 2).

### TSA

**Figure 6:**
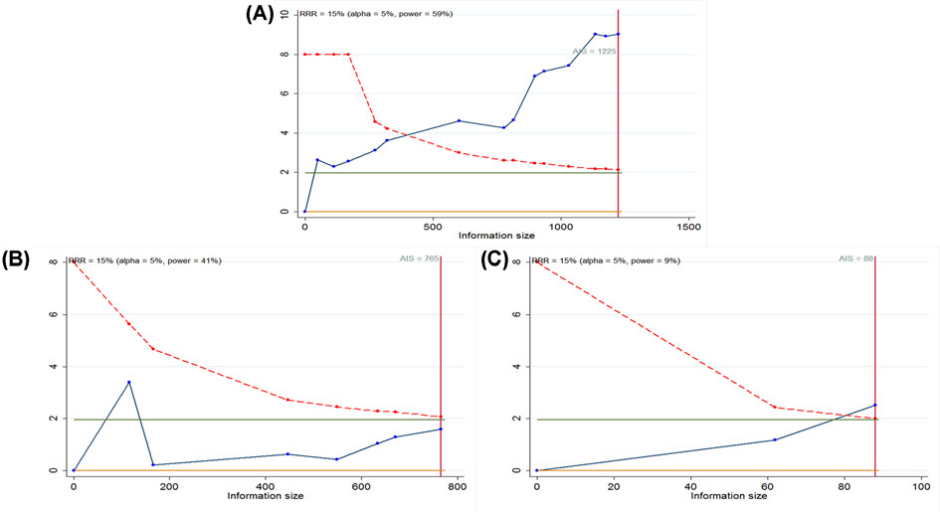
Trial sequential analysis (TSA) for the primary outcomes TSA for OS (**A**), DFS (**B**), and RFS (**C**).

The cumulative Z-curve (blue line) was found to cross both the traditional boundary line and the trial sequential monitoring boundary (red line), and the cumulative information reached the required information size (RIS) for OS and RFS (Figure 6A,C). Thus, additional studies for OS were not required. Additional studies for RFS were necessary due to the insufficient number of samples; however, the cumulative Z-curve (blue line) crosses the traditional boundary line rather than crossing the trial sequential monitoring boundary (red line), while the cumulative information reaches the RIS for DFS (Figure 6B).

### Validation by independent TCGA digestive system cancer datasets

**Figure 7:**
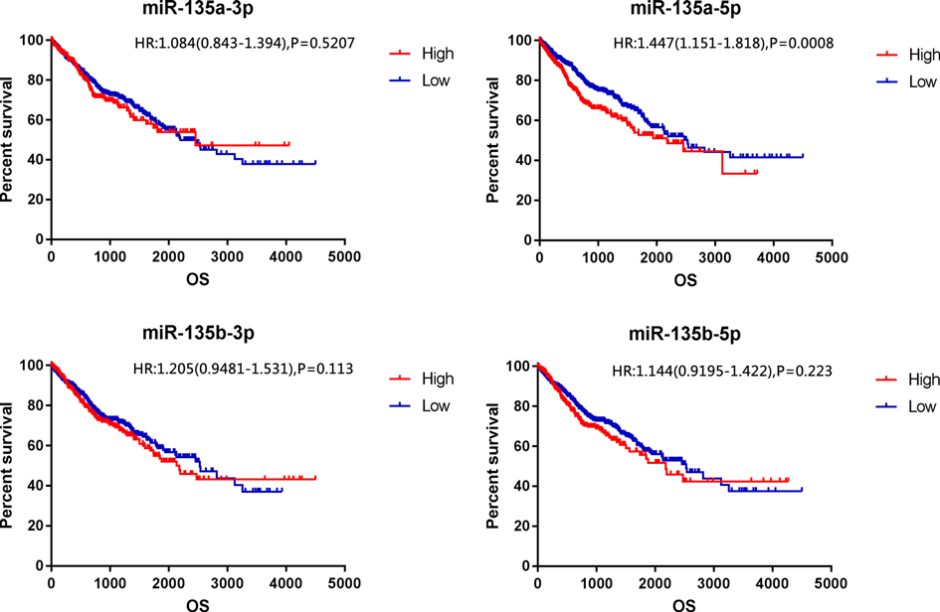
The relationship between miR-135 subtypes and prognosis of digestive system cancers using TCGA data

To verify the meta-analysis results, we performed an analysis of TCGA database regarding the level of miR-135 expression and corresponding survival data. This analysis included seven types of cancer: cholangiocarcinoma, colon adenocarcinoma, esophageal carcinoma, pancreatic adenocarcinoma, liver HCC, rectum adenocarcinoma, and stomach adenocarcinoma. Moreover, the results showed that high miR-135 expression in the tissues was associated with a poorer OS for digestive system cancers (HR: 1.217; 95% CI: 1.083–1.368; *P*=0.002). And, the relationship between miR-135 subtype and prognosis of digestive system cancers is shown in Figure 7. In the analysis of single tumor types, we found that high miR-135 expression was associated with a poor OS for colon adenocarcinoma (HR: 1.526; 95% CI: 1.051–2.214; *P*=0.026), pancreatic adenocarcinoma (HR: 1.403; 95% CI: 1.042–1.888; *P*=0.026), liver HCC (HR: 1.654; 95% CI: 1.221–2.241; *P*=0.001), and stomach adenocarcinoma (HR: 1.303; 95% CI: 1.010–1.681; *P*=0.042) (Table 3). In the other tumor types, miR-135 expression was not associated with the prognosis of patients with cholangiocarcinoma, esophageal carcinoma, and rectum adenocarcinoma (Table 3). Therefore, TCGA results were consistent with the findings of our meta-analysis.

**Table 3:** HRs and corresponding 95% CIs of miR-135 overexpression in digestive system cancers based on TCGA datasets

Cancer type	Sample sizes	miR-135 assay	HR (95% CI)	*P*-value
Digestive system cancer	1488	IlluminaHiseq	1.217 (1.083–1.368)	0.002
Cholangiocarcinoma	36	IlluminaHiseq	1.432 (0.808–2.539)	0.218
Colon adenocarcinoma	249	IlluminaHiseq	1.526 (1.051–2.214)	0.026
Esophageal carcinoma	182	IlluminaHiseq	1.344 (0.967–1.869)	0.078
Pancreatic adenocarcinoma	369	IlluminaHiseq	1.403 (1.042–1.888)	0.026
Liver HCC	177	IlluminaHiseq	1.654 (1.221–2.241)	0.001
Rectum adenocarcinoma	88	IlluminaHiseq	2.284 (0.893–5.482)	0.085
Stomach adenocarcinoma	387	IlluminaHiseq	1.303 (1.010–1.681)	0.042

## Discussion

This meta-analysis represents the first systematic evaluation of the relationship between the level of miR-135 expression and the prognosis of patients with digestive system cancers. A total of 17 studies consisting of 1470 patients were included in this meta-analysis. Our results revealed that a greater level of miR-135 expression was associated with a poorer OS and RFS in patients with digestive system cancers. Stratified analyses were performed, and significant results pertaining to the relationship between miR-135 and a poor OS were some of tumor type, digestive function, biomarker subtype, publication year, sample size, the method of HR extraction, and quality score. There was no significant heterogeneity and publication bias in this pooled result, which suggested that our results were reliable. In addition, the TSA indicated that the sample size was sufficient and the OS results were reliable due to the incalculable probability of false-positive reports in the FPRP analysis. In addition, the results were verified using TCGA database analysis. These findings suggested that miR-135 overexpression is indicative of a poor prognosis and higher recurrence in patients with digestive system cancers. However, the results required further validation for DFS and RFS due to the high probability of false-positive reports in the FPRP analysis.

Stratified analyses showed that article factors, including publication time, sample size, studies quality, and methods of HRs, had no effect on the association between miR-135 expression and the OS of patients with digestive system cancers. Although mature miR-135a and miR-135b only differ regarding one nucleotide, this did not impact their prognostic effect because both of the miRNAs target the same genes. In the subgroup analysis, there was significant heterogeneity associated with the gastric cancer group due to the two contrary results of the incorporated studies. While Cheng et al. [31] found that decreased miR-135 expression was indicative of a poor OS, a study by Yan et al. [15] reported the opposite findings. However, TCGA database analysis revealed that miR-135 expression had prognostic significance for gastric cancer. Thus, future large-scale studies should be performed to verify this speculation and consolidate the pooled results. In addition, we used the data from TCGA datasets to validate the meta-analysis results. Similar results were found for pancreatic, colon, and hepatocellular cancers.

An increasing number of studies have shown that the deregulation of miRNA function is associated with an increasing number of human diseases, particularly cancer. Moreover, miRNA occupies an important position in regulating cancer proliferation, growth, invasion, and migration. Conventional non-invasive serological biomarkers, including carcinoembryonic antigen (CEA) and cancer antigen 19-9 (CA19-9), can be screened using blood tests that are currently being applied in the clinic. However, these biomarkers lack specificity for the early diagnosis of digestive system cancer [42,43]. Although some studies have found that CEA and CA19-9 could predict the prognosis of patients with digestive system cancer, including CRC and gastric cancer [44,45], the positive rate is too low. In contrast, miRNAs in digestive cancer patients may represent promising biomarkers that are stable and reproducible in both the peripheral blood and tissues. In the peripheral blood, circulating miRNAs primarily bind to proteins or are capsulated in exosomes [46]. Although measuring plasma or serum miRNA is non-invasive, the level of circulating miRNAs is susceptible to perturbations due to blood cells or hemolysis compared with tissue miRNA [47]. In addition, it is not difficult to measure tissue miRNA in patients after surgery. Moreover, the detection of tissue miRNA was more accurate, with fewer influencing factors. It was convincing that changes in miRNA expression and patient survival was consistent in both the peripheral blood and tissues. However, the results may differ on occasion; for example, when Zhang et al. [48] elucidated the prognostic value of miR-20a in human cancers, high expression of both circulating and tumorous miR-20a were associated with an unfavorable outcome only in gastrointestinal cancers. In this meta-analysis, all the included studies were related to tissue miRNAs with reliable results.

Kawaguchi et al. [49] proposed the use of miRNA as a next-generation biomarker for digestive system cancers. In addition, the authors thought that several miRNAs should be used in combination to evaluate the prognosis of one or more cancers. Recently, a database analysis demonstrated that a panel of miRNAs, including miR-135, was associated with a poor prognosis for both pancreatic and esophageal cancers [16,17,50]. Therefore, although meta-analyses have shown that several miRNAs, including miR-17-5p [51] and miR-133a [52], could predict the prognosis of digestive system tumors, a meta-analysis was required to assess the relationship between miR-135 and digestive system cancers. Moreover, miR-135 has been found to be involved in mediating the metabolic stress response in cancer. For example, miR-135 can modulate critical signaling pathways (e.g., JAK/STAT [14], P38MAPK/NF-κB [53], PTEN/PI3k/AKT [54], and RERG [34]) that indirectly regulate metabolism. Altered metabolism has long been recognized as a central hallmark of cancer. Yang et al. [12] established a key role for miR-135 in regulating glycolysis *in vivo* and found that miR-135 suppressed glycolysis and promoted PC cell adaptation to metabolic stress by targeting phosphofructokinase-1. In addition, a study by Wang et al. [14] found that miR-135 increased the levels of phosphorylated key kinases in the JAK/STAT pathway and functioned as a tumor promoter by targeting TRIM16. Furthermore, the deregulation of miR-135 function has been associated with both breast cancer [13] and Hodgkin lymphoma [55]. These findings provide evidence for the function of miR-135 as a potential prognostic factor and treatment for tumors.

There were some limitations associated with the present research. First, due to insufficient data, only 17 studies were recruited, comprising a total of 1470 patients in this meta-analysis. Moreover, some studies that yielded negative results were generally not published. Due to these factors, some subgroups only included one study in the subgroup analysis. Therefore, this result may be an outlier because the data were insufficiently comprehensive. Second, most of the included patients were Asian, which might reduce the universal applicability of the results. Thus, additional large-scale studies should be performed among different ethnicities. Third, the cut-off value of miR-135 was variable in different studies, which did not reach a consensus value in this meta-analysis. Fourth, HRs with associated 95% CIs and *P*-value extracted from the curve differed from the actual value because some of the studies did not provide HRs with 95% CIs.

In conclusion, the results of this meta-analysis indicate that miR-135 expression might be a novel prognostic factor for the survival of patients with digestive system malignancies. Further studies with a large-scale sample size should be performed to identify the association between miR-135 expression and digestive system cancer prognosis.

## Abbreviations

CA19-9: cancer antigen 19-9
CEA: carcinoembryonic antigen
CRC: colorectal cancer
DFS: disease-free survival
ESCC: esophageal squamous cell carcinoma
FPRP: false-positive report probability
HCC: hepatocellular carcinoma
HR: hazard ratio
miR-135: microRNA-135
OS: overall survival
PC: pancreatic cancer
RFS: recurrence-free survival
RIS: required information size
TCGA: The Cancer Genome Atlas
TSA: trial sequential analysis
95% CI: 95% confidence interval
